# *Poa magellensis* (Poaceae), a new species from Central Apennine (Italy)

**DOI:** 10.3897/phytokeys.144.49971

**Published:** 2020-04-03

**Authors:** Fabio Conti, Luciano Di Martino, Fabrizio Bartolucci

**Affiliations:** 1 Scuola di Bioscienze e Medicina Veterinaria, Università di Camerino – Centro Ricerche Floristiche dell’Appennino, Parco Nazionale del Gran Sasso e Monti della Laga, San Colombo, 67021 Barisciano (L’Aquila), Italy Università di Camerino San Colombo Italy; 2 Ufficio Monitoraggio e Conservazione della Biodiversità vegetale, Parco Nazionale Majella, Via Badia 28, 67039 Sulmona (L’Aquila), Italy Ufficio Monitoraggio e Conservazione della Biodiversità vegetale Sulmona Italy

**Keywords:** Abruzzo, endemic, Italy, Majella National Park, new species, *
Poa
*, Poaceae

## Abstract

A new species, *Poa magellensis***sp. nov.**, is described and illustrated based on collections from the Majella Mountains in Central Apennine, Italy. It is morphologically similar to *P. ligulata* Boiss., but can be distinguished by green leaves, the basal ones adaxially lightly scabrous or sparsely hairy, longer ligules particularly in the upper leaves, panicles denser with primary branches sub-erect to erect, glumes with broader scarious margin, more acute glumes and lemmas, lemmas and paleas longer, rachilla hairy, calluses usually with a crown of hairs or shortly webbed, caryopses longer. A distribution map of the species is also presented.

## Introduction

The genus *Poa* L. (Poaceae, Pooideae, Poeae, Poinae), one of the largest genera of grasses, has a cosmopolitan distribution, principally in temperate regions of both hemispheres and in mountainous regions of the tropics (Soreng et al. 2017; Peterson and Soreng 2018). It is a monophyletic genus and comprises approximately 550 annual and perennial species (Soreng 1990; Gillespie et al. 2007, 2008; Soreng et al. 2017). Based on molecular phylogenetic studies, *Poa* has been divided into five subgenera: P. subg. Ochlopoa (Asch. & Graebn.) Hyl., P. subg. Poa, P. subg. Pseudopoa (K.Koch) Stapf, P. subg. Stenopoa (Dumort.) Soreng & L.J.Gillespie and P. subg. Sylvestres (V.L.Marsh ex Soreng) Soreng & L.J.Gillespie (Gillespie et al. 2007, 2008; Soreng et al. 2010; Giussani et al. 2016). As concerns Italy, the genus *Poa* is represented by 29 taxa (species and subspecies) (Bartolucci et al. 2018). Recently, two other species have been added to the flora of Italy: *P. jubata* A.Kern. (Brullo et al. 2019) and *P. ligulata* Boiss. (Conti et al. 2019). This latter taxon belonging to P. subg. Ochlopoa sect. Alpinae (Hegetschw. ex Nyman) Stapf, occurs in Spain and NW Africa (Buschmann 1942; Ortega-Olivencia and Devesa 2018) and recorded in France (Greuter and Raus 2005) by mistake (Tison and de Foucault 2014). Its recent discovery in Central Apennine was considered as a confirmation for Italy; in fact, this species had already been collected by G. Rigo in Central Apennine on Majella Mountains “*Iter in Aprutio anno 1905. Poa magellensis mihi, proxima P. ligulata Bss. In pascuis alpinis di M. Amaro, calc. 2600 m, Jul. exeunte G. Rigo* ” (BP). Buschmann (1942) quoted the specimen preserved at BP and listed *P. magellensis* Rigo *in sched.* (this name was never validly published) as a doubtful synonym of *P. ligulata* and regarded the collecting site of the specimen doubtful. After this record the species was no longer reported for Italy until the discovery by Conti et al. (2019). The specimen traced in BP and others collected by us in Majella were provisionally attributed to *P. ligulata* (Conti et al. 2019). Indeed, on the basis of a preliminary morphological analysis, the population from Majella showed peculiar features that led us to compare it with those from Spain and Morocco referable to *P. ligulata*.

## Materials and methods

This study is based mainly on field surveys, on an extensive analysis of relevant literature, and on careful examination of herbarium specimens preserved at APP, BC, BP, SALA (acronyms follow Thiers 2019). In order to investigate the morphological variability of *Poa ligulata* and to correctly classify the population from Italy, morphological analyses were carried out on 40 selected specimens including *Poa* from Central Apennine (20 specimens) and *P. ligulata* from Spain and Morocco (20 specimens). The individuals were studied measuring 20 quantitative characters (see Table 1). Other qualitative characters were studied: shape of ligule, shape of glume, shape of lemma, shape of palea, rachilla hairness, callus hairness. Morphological observations and measurements were conducted on living and dried (primarily) specimens. All morphological characters were observed and photographed with a Leica MZ16 stereoscopic microscope and a Canon S50 camera.

**Table 1.** Morphological quantitative characters studied.

Character
culm height (mm)
number of culm nodes
blade length (basal leaf) (mm)
blade width (basal leaf) (mm)
ligule length (basal leaf) (mm)
ligule length (upper leaf) (mm)
panicle length (mm)
panicle width (mm)
rachis diameter (mm)
spikelet length (mm)
spikelet width (mm)
number of flowers in each spikelet (mm)
lower glume length (mm)
upper glume length (mm)
scarious margin width of glume (mm)
lemma length (mm)
length of hair strip on the lemma keel (mm)
palea length (mm)
anther length (mm)
caryopsis length (mm)

## Taxonomy

### Poa magellensis

Taxon classificationPlantaePoalesPoaceae

F.Conti & Bartolucci sp. nov.

D5816944-0FB5-5CD9-BCF9-2557FDA5E4D7

urn:lsid:ipni.org:names:77208269-1

Figs 1 , 2 , 3

#### Type.

Italy. Abruzzo, Fara S. Martino (Chieti), Majella, M. Acquaviva (WGS84 42°06'11.1"N, 14°07'55.9"E), 2720 m, pendii rupestri, 30 Jul. 2019, *F. Conti, L. Di Martino & V. Di Cecco s.n.* (holotype: APP 65502; isotype: APP 65501).

#### Diagnosis.

*Poa magellensis* differs from *P. ligulata* by: basal leaves adaxially lightly scabrous or sparsely hairy vs glabrous, longer ligules particularly in the upper leaves (2.5)4–6.9(9) vs. (1.8)2–5(6) mm long, panicles denser with primary branches sub-erect to erect, glumes with broader scarious margin 0.1–0.4(0.5) vs. 0–0.2 mm wide, more acute glumes and lemmas, longer lemmas (2.7)2.9–3.5(3.6) vs. (2.1)2.3–2.9(3) mm long, longer paleas (2.2)2.5–3.1(3.2) vs. 2–2.8(3) mm long, rachilla hairy vs glabrous, calluses usually with a crown of hairs or shortly webbed vs glabrous, longer caryopses 1.7–2.2 vs. 1.4–1.7 mm long.

#### Description.

Perennials; without horizontal or downward tending cataphyllous shoots, densely tufted, bicolour, green and white because of the brightness of large exerted ligules; tillers erect or ascending, intravaginal. *Culms* 20–150 mm tall, 0.25–0.5 mm in diameter, erect to ascending slender, terete, smooth, weakly sulcate, nodes (1)2 exerted, thickened at the base with old leaf-sheaths. *Leaves* green, leaves-sheaths terete, smooth, glabrous, ribbed; collars smooth, glabrous; blades of basal leaves (8)8.6–19.4(20) mm long, 0.8–1.5(1.6) mm wide, linear, usually folded, abaxially rough, margins lightly scabrous, adaxially lightly scabrous or sparsely hairy, prow-tipped, blades strongly graduated or reduced distally, blades of uppermost leaves 3.5–12 mm long, ligules of the basal leaves (4)4.7–10.2(12) mm long, smooth, glabrous, whitish-pearly, lacerate, apices acuminate, decurrent on the sheaths; ligules of the uppermost leaves (2.5)4–6.9(9) mm long. *Panicles* compact, narrowly ellipsoid, (11)11.6–22.4(23) mm long, 5–10(13) mm wide, dense, with 3–7 nodes, rachis with 1–2(–3) branches per node; primary branches sub-erect to erect, sulcate or few-angled, scabrous 0.1–0.3 mm in diameter, longest branches up to 5 mm, with 1–3 spikelets, pedicels 0.1–4 mm. *Spikelets* 3.2–5(5.4) mm long, (1.3)1.4–3.2(3.5) wide, laterally compressed; bulbifery absent, violaceous and green, not pruinose, florets 2–4(5); rachilla hairy to sparsely hairy. *Glumes* subequal (1–)3 veined, not reaching lemma apices, lanceolate, with scarious margin 0.1–0.4(0.5) mm, glabrous, distinctly keeled, keels moderately scabrous in the distal part, apices sharply-acute; lower glumes (2.1)2.2–3.3(3.4) mm long; upper glumes (2.4)2.5–3.6 mm long; calluses with a crown of hairs (0.1–0.3 mm) or shortly webbed; *lemmas* (2.7)2.9–3.5(3.6) mm long, 1–5 weakly veined, lanceolate, violaceous or sometimes green, with scarious margin broader in the distal part, distinctly keeled, keels short villous in proximal part (1–2 mm), scaberulous along distal keel and sparsely in the upper sides, apices acute; paleas (2.2)2.5–3.1(3.2) mm long, scabrous along the keels, between keels glabrous. *Anthers* 1–2 mm long. *Caryopses* 1.7–2.2 mm long.

**Figure 1.**
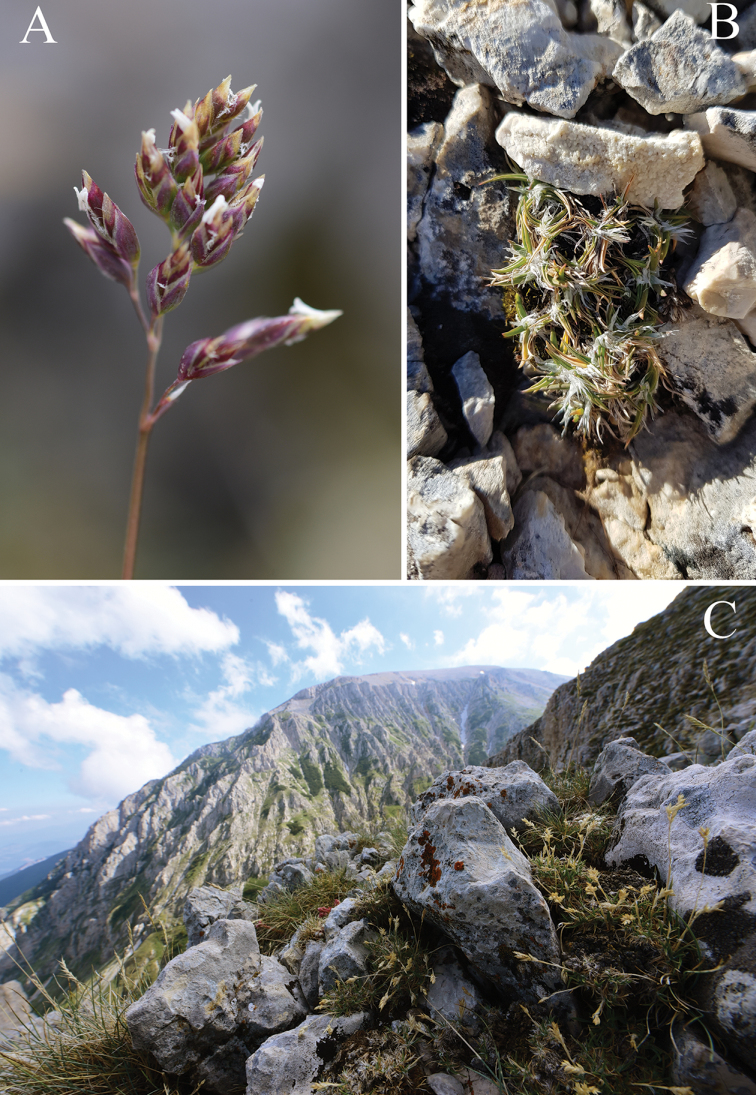
*Poa magellensis* F.Conti & Bartolucci, sp. nov. **A** panicle (Cima delle Murelle, Abruzzo, Italy; photo by F. Conti) **B** tuft bicoloured, green and white because of the brightness of the large exerted ligules (Cima delle Murelle, Abruzzo, Italy; photo by F. Bartolucci) **C** species habitat (Cima delle Murelle, Abruzzo, Italy; photo by F. Conti).

**Figure 2.**
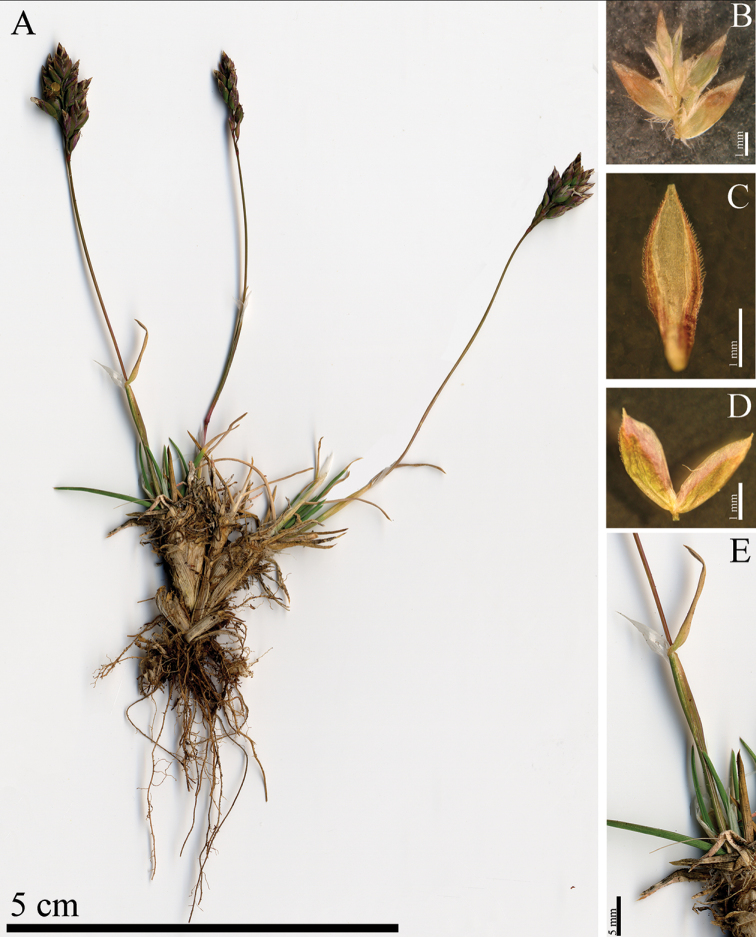
*Poa magellensis* F.Conti & Bartolucci, sp. nov. **A** habit **B** spikelet without glumes **C** palea **D** glumes **E** ligules.

**Figure 3.**
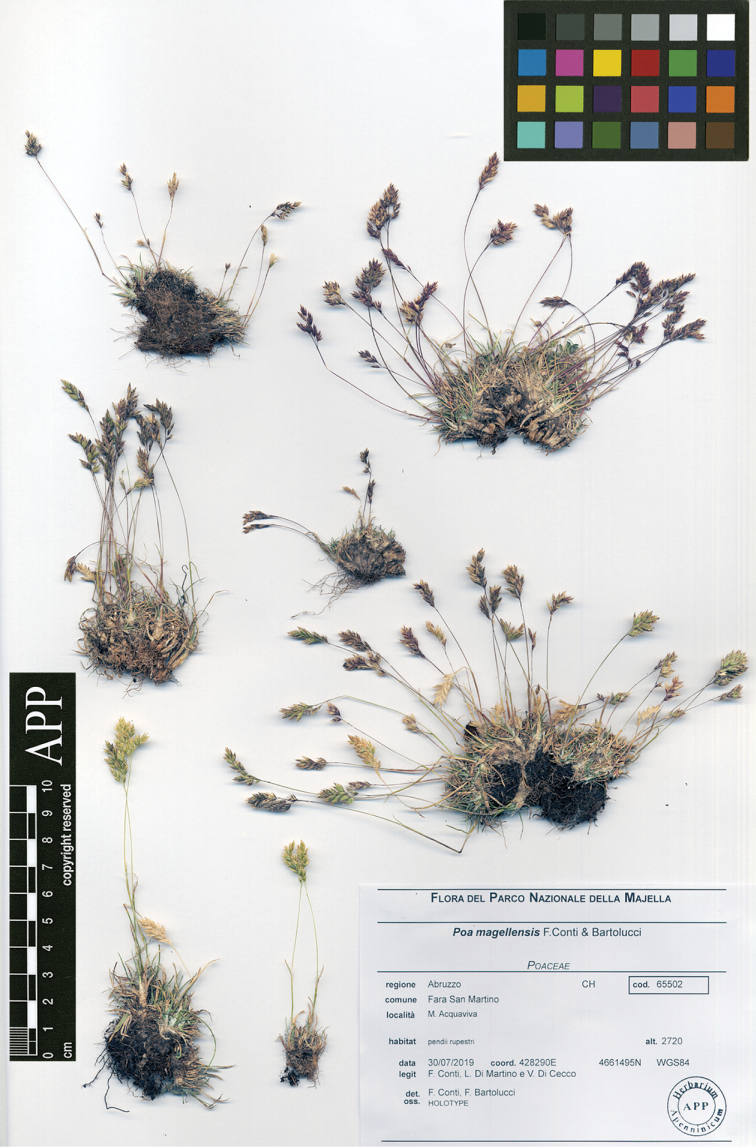
Holotype of *Poa magellensis* F.Conti & Bartolucci (APP, reproduced with permission of the Herbarium, Centro Ricerche Floristiche dell’Appennino, Italy).

#### Distribution and habitat.

**Figure 4.**
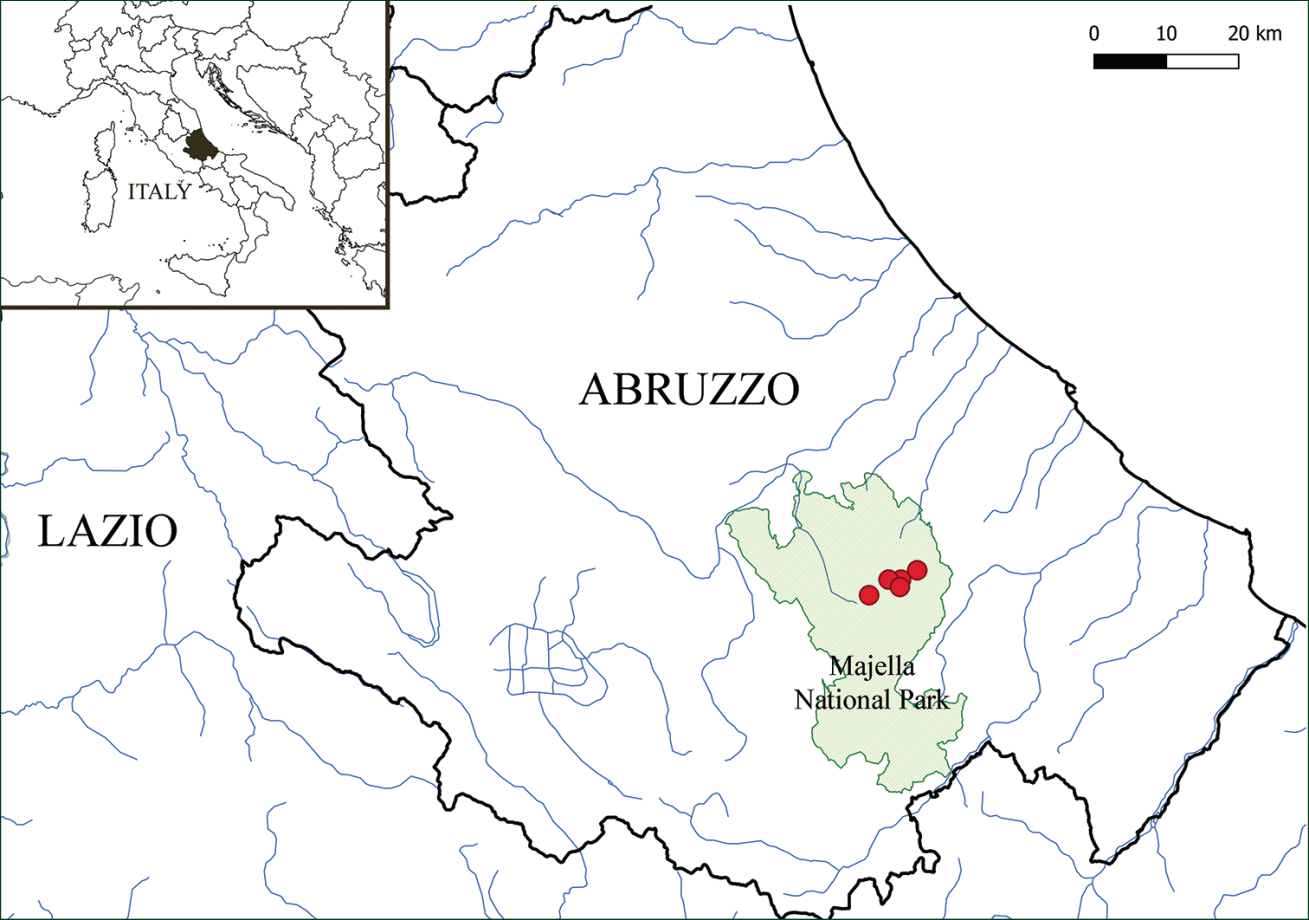
Map showing distribution of *Poa magellensis* F.Conti & Bartolucci, sp. nov. in Central Apennine, Abruzzo (Italy).

*Poa magellensis* is endemic to Majella Mountains (Mt. Amaro, Mt. Focalone, Mt. Acquaviva, between Mt. Focalone and Mt. Acquaviva, Cima delle Murelle) in Central Apennine (Italy). It grows on limestone rocky slopes from 2200 up to 2730 m a.s.l. (Fig. 4).

#### Phenology.

Flowering in July, fruiting July to August.

#### Chromosome number.

A single population (Majella, Cima delle Murelle) of *P. magellensis* is resulted diploid with 2n = 14 chromosomes (Astuti et al. 2019 under the name *P. ligulata*).

#### Conservation status.

The populations of *P. magellenis* are included in NATURA 2000 network within the Site of Community Interest “IT140203 Majella” in the Majella National Park. The extent of occurrence (EOO) is 6.86 km^2^ calculated with minimum convex hull polygon in QGIS and area of occupancy (AOO) is 16 km^2^ calculated with a 2×2 km cell fixed grid. The species occurs in only one location (definition according to IUCN 2019), but the population is not declining, and there are no extreme fluctuations. According to IUCN criterion B (2019) we propose to include *P. magellensis* in the following category: Near Threatened (NT).

#### Etymology.

The specific epithet of the new species is derived from the type locality, Magella Mountains (currently Majella Mountains).

#### Taxonomic notes.

The new species *P. magellensis* is similar to *P. ligulata* but can be distinguished by several characters as shown in Table 2.

**Table 2.** Comparison of the key features of *Poa ligulata* and *P. magellensis*. Quantitative continuous characters are expressed in mm and are reported as mean ± standard deviation and 10−90 percentiles (extreme values in brackets). For quantitative discrete cardinal characters, 10−90 percentiles are given (extreme values in brackets).

Character	*Poa ligulata*	*Poa magellensis*
Leaves	green or glaucous	green
adaxial blade (basal leaf)	glabrous	lightly scabrous or sparsely hairy
blade width (basal leaf)	(0.9)1–2.5(2.7)	0.8–1.5(1.6)
	1.53±0.49	1.07±0.23
ligule length (upper leaf)	(1.8)2–5(6)	(2.5)4–6.9(9)
	3.35±1.13	5.35±1.44
panicle width	(8)8.5–20	5–10(13)
	13.81±4.22	7.6±2.13
number of flowers in each spikelet	3–6(7)	2–4(5)
upper glume length	2–2.8(3)	(2.4)2.5–3.6
	2.45±0.25	3.08±0.3
scarious margin width of glume	0–0.2	0.1–0.4(0.5)
	0.08±0.07	0.28±0.11
apex of glume	acute	sharply-acute
lemma length	(2.1)2.3–2.9(3)	(2.7)2.9–3.5(3.6)
	2.6±0.23	3.23±0.24
palea length	2–2.8(3)	(2.2)2.5–3.1(3.2)
	2.37±0.28	2.87±0.23
caryopsis length	1.4–1.7	1.7–2.2
	1.52±0.12	1.94±0.16
hairs on rachilla	absent	present
hairs on callus	absent	present

#### Additional specimens examined.

***Poa magellensis*** F.Conti & Bartolucci (paratypes): Italy. Abruzzo: in pascuis alpinis di M. Amaro, calc., m. 2600, Jul exeunte, *G. Rigo s.n.* [Iter in Aprutio anno 1905] (BP); Majella, sotto l’anfiteatro delle Murelle e il Fusco, verso il Blockhaus, Pennapiedimonte (Chieti), pendii rupestri, 2250–2500 m, 04 Aug. 1991, *F. Conti s.n.* (APP 12455); Majella, dal Blockhaus al Focalone, Caramanico Terme (Pescara), pendii rupestri, 2350 m, 30 Jul. 2009, *F. Conti, L. Gubellini & R. Soldati s.n.* (APP 59356); salendo a Cima delle Murelle in loc. La Carozza, Pennapiedimonte (Chieti), rupi e pendii rupestri, 2300 m, 02 Sep. 2011, *F. Bartolucci & F. Conti s.n.* (APP 59212, 59214); Majella, Cima delle Murelle, Pennapiedimonte (Chieti), pendii rupestri, 2250 m, 17 Jul. 2019, *F. Conti & L. Di Martino s.n.* (APP 65242, 65243, 65244, 65245, 65246, 65247, 65248, 65249, 65250, 65251, 65252, 65254, 65253, 65255). ***Poa ligulata*** Boiss.: Morocco. c38 km from Chefchaouen, 14 km above Bab Taza on track to Djbel Talamssemtane (35°9'N, 5°12'W), forest of *Abies maroccana* and *Cedrus atlantica*, on limestone, 1765–1900 m, 26 Jun. 1992, *Achhal et al. n. 64.2260* [Optima Iter Mediterraneum V] (APP 45892; SALA 144759); Tanger-Tétouan: Bab Taza, pr. Refugio del Jbel Lakraa, 35°8'11,7"N, 5°8'13,6"W, 1693–2000 m, substrato calizo dolomitico, 16 Jun. 2008, *S. Andrés et al. n. AQ2695* (SALA 159115); Spain. Aznattin W. (Macizo de Magina pr. Jaén) 1710 m. riscas, 20 Jun. 1926, *Cuatrecasas s.n.* (BC-70613); Carceles in decliv. NW, rupestr. 1950 m. alt., 17 Jun. 1826, *Cuatrecasas s.n.* (BC-70612); Sierra Tejeda, Málaga, 10 Jun. 1919, *Gros s.n.* (BC-70615); Cadiz: Grazalema, 30 Jun. 1925, *Font i Quer & Gros s.n.* (BC-914825); Baetica: in graminosis l. Cerrecillos del Sabinal dicto, montis Sierra de Gador (Almeria), ad 2000 m alt., 27 May 1921, *Gros n. 108* [Flora Iberica selecta Cent. II] (BC-86523,BC-61436, BC-86524); *ibidem*, *Gros s.n.* (BC-70614); Cerro de la Laguna, Sierra de Cazorla, 1550 m, (J) WG0295, in locis lapidosis cacuminalis, 25 May 1981, *A.M. Hernández s.n.* (BC-641787); Teruel: Palomar de Arroyos, port de Sant Just, entre Escucha i Palomar de Attoyos, UTM 30T 687830 4515699, 1486 m, 02 Jun. 2010, *S. Pyke & M. Aixart s.n.* (BC-904950); Sierra de Javalambre (España, prov. Teruel), 30S XK 74, alt 1850 m, pastizal subnitrófilo de alta montaña, *Festuco-Pojon ligulatae* Rivas Goday & Rivas Martínez, 01 Jun. 1985, *M. Costa et al. n. 13853* (SALA 83393); La Rioja: Turruncún, Sierra de Préjano, Peña Isasa, 30T WM7068, 1450 m, en crestones y pequeñas repisas con suelo esquelético, caracterizando los céspedes xerofiticos del *Festuco-Pojon ligulatae* Rivas Goday & Rivas Martínez 1966, 29 Jun. 1988, *Amich, Fdez. Diez & Sanchez Rodriguez n. 97* [Exsiccata Selecta Flora Ibericae] (SALA 83963, 45290); Villafeliz de Babia (León), 20 May 1983, *J. Andrés & Fllamas s.n.* (SALA 68424); Granada: Sierra Nevada, Puerto de la Ragua, suelos pedregosos, 17 Jul. 1971, *Ladero & E. Valdés s.n.* (SALA 64021); Valporqueto (Espagne, prov León, Cordillera Cantábrica), alt. 1250 m, pâturages pierreux dans la zone subalpine cantabrique, fissures et replats des rochers calcaires, communautés du *Festuco-Pojon ligulatae*, 06 Jul. 1980, *J.M. Losa Quintana n. 11026* (SALA 70101); Segovia: Navares de las Cuevas, Peñacuero, 08 Jun. 1985, *Rico, T. Romero & Sánchez Rodriguez s.n.* (SALA 49384); Pastizales pedregosos en el Cerro de San Cristobal, Grazalema (Cádiz), Jun. 1961, *Borja s.n.* (SALA 1747); Ciudad encantada (Cuenca), 13 May 1977, *Fernández Diez, Rico, Amich & Sánchez s.n.* (SALA 11599); Guadalajara: Saúca, 30T WL 4041, 1150 m, páramo calizo con *Genista pumila*, 22 Jun. 1987, *L. Villar & P. Montserrat s.n.* (SALA 125139); Cádiz, Grazalema base del Cerro de San Cristobal, 23 May 1966, *E.F. Galiano s.n.* (SALA 22260); Teruel: nacimiento del rio Tajo, 15 Jun. 1982, *Rico & Sánchez s.n.* (SALA 32128); León: Ponferrada, Peñalba de Santiago, en calizas, 25 Jun. 1984, *S. Castroviejo, J.L. Fernández Alonso, Gonzalo & Valdes s.n.* (SALA 103309); Prado Martín, Penyagolosa prov. Castellón, 1550 m, *Poo-Festucetum hystricis*, 20 Jun. 1980, *I. Soriano s.n.* (SALA 71015); Granada: Sierra Nevada, Barranco de San Juan, en suelos pedregosos, 3200 m, 10 Jul. 1980, *M. Ladero, López-Guadalupe & Molero* (SALA 61347); Castellon: El Toro (L’Alt Palància), la Halmarja, 30S XK82, 1500 m, patizales ralos de Minuartio-Pojon, 18 May 1984, *A. Aguilella n. 15828* (SALA 90109); Segovia: villar de Sobrepeña, Cerro del Valdemuela, 15 May 1983, *T. Romero s.n.* (SALA 40608); Segovia; Sepúlveda, 01 Jun. 1986, *X. Giraldez & T. Romero s.n.* (SALA 41703); Segovia: Prádena, Peña Corva, 02 Jun. 1984, *T. Romero s.n.* (SALA 40607); Burgos: Hontorio de la Cantera, 30T VM 4769, 950 m, frecuente, encinar, calizas de cantera subterraneas, 16 May 2004, *J.L.B. Alonso s.n.* (SALA 124549); Cadiz: Sierra de Grazalema, 12 Jun. 1976, *A.M. Hernandez s.n.* (SALA 31879).

##### Key to identification of species of Poa sect. Alpinae in Europe (from Edmondson 1980 modified)

**Table:** 

1	Lemma hairy between veins	**2**
–	Lemma glabrous between veins	**4**
2	Culm below the panicle 0.7–1 mm in diameter; leaves 4–10 cm long; lower cauline and basal leaves with a short, ± truncate ligule; panicle ± pyramidal	***P. alpina***
–	Culm below the panicle 0.2–0.5 mm in diameter; leaves (1.5)2.5–6 cm long; cauline and basal leaves with an elongate, acute ligule; panicle ± ovoid	**3**
3	Leaves 2–4.5 mm wide, flat to weakly folded	***P. badensis***
–	Leaves 1.5–2.5 mm wide, canaliculate to strongly folded	***P. molinerii***
4	Stem (15)20–30(40) cm; panicle 3.5–5 cm, ellipsoid-oblong	***P. media***
–	Stem 3.5–20(30) cm; panicle 1–5 cm, ellipsoid, ovoid to pyramidal	**5**
5	Ligule of the basal leaves 1–2 mm, hyaline	**6**
–	Ligule of the basal leaves 2.5–12 mm, milky white	**7**
6	Lemma callus glabrous	***P. thessala***
–	Lemma callus woolly	***P. pumila***
7	Leaves green, adaxially lightly scabrous or sparsely hairy, ligule of the upper leaves (2.5)4–6.9(9) mm, panicle narrowly ovoid, glume with scarious margin 0.1–0.4(0.5) mm, glume sharply acute, lemma (2.7)2.9–3.5(3.6) mm, rachilla hairy, callus usually with a crown of hairs or shortly webbed, caryopsis 1.7–2.2 mm	***P. magellensis***
–	Leaves green or glaucous, adaxially glabrous, ligule of the upper leaves (1.8)2–5(6) mm, panicle ovoid to pyramidal, glume without or with scarious margin up to 0.2 mm, glume acute, lemma (2.1)2.3–2.9(3) mm, rachilla glabrous, callus glabrous, caryopsis 1.4–1.7 mm	***P. ligulata***

## Supplementary Material

XML Treatment for Poa magellensis
